# The complete mitochondrial genome of *Trichopria drosophilae* (Hymenoptera: Diapriidae)

**DOI:** 10.1080/23802359.2020.1775521

**Published:** 2020-06-08

**Authors:** Zhang Xian, Zhongqiu Pan, Jiani Chen, Jiachen Zhu, Sicong Zhou, Lan Pang, Min Shi, Xuexin Chen, Jianhua Huang

**Affiliations:** a Institute of Insect Sciences, College of Agriculture and Biotechnology, Zhejiang University, Hangzhou, China; b Ministry of Agriculture Key Lab of Molecular Biology of Crop Pathogens and Insect Pests, Zhejiang University, Hangzhou, China; c Key Laboratory of Biology of Crop Pathogens and Insects of Zhejiang Province, Zhejiang University, Hangzhou, China; d State Key Lab of Rice Biology, Zhejiang University, Hangzhou, China

**Keywords:** *Trichopria drosophilae*, parasitoid, mitochondrial genome

## Abstract

*Trichopria drosophilae* (Hymenoptera: Diapriidae) is an important pupal endoparasitoid of *Drosophila* species, which has been found to be an ideal biocontrol agent to *D. suzukii*. In this study, the complete mitochondrial genome of *T. drosophilae* (GeneBank accession number: MN966974) was sequenced using Illumina HiSeq X Ten system. The mitochondrial genome is 16,375 bp long and comprises 13 protein-coding genes, 22 transfer RNA genes and 2 ribosomal RNA genes. Among them, 24 genes are in majority strand, while the others are in minority strand. The nucleotide composition of A, G, C, T is 44.9%, 6.4%, 5.6%, 43.2% respectively. We also performed a phylogenetic analysis with other known mitochondrial genomes within four families that have been shown to parasitize drosophilid species. The result shows that *T. drosophilae* is closely related to *Ismarus sp.*

*Trichopria drosophilae* is a cosmopolitan pupal endoparasitoid, whose distribution was widely ranging from Asia, Europe to America. Recently, *T. drosophilae* appeared to be a perfect biological control agent against *Drosophila* pests, especially to *D. suzukii*, a worldwide invasive and destructive pest of soft and thin-skinned fruit crops (Yi et al. 2020). Lots of work have been done in the field of biological traits and development of *T. drosophilae* to improve the biocontrol efficacy (Chen et al. 2018; Zhou et al. 2019). However, little has been done about their phylogenetic analysis and evolution.

*T. drosophilae* was collected from traps baited with grape fruits on May 2016 at Zijingang Campus (30.29°N, 120.08°E), Zhejiang University, Hangzhou, Zhejiang, China. After sampling, the specimen (ZJUHJH_002) was stored in 100% ethanol and kept in the Parasitic Hymenoptera Collection Institute of Insect Sciences, Zhejiang University. The DNA of *T. drosophilae* was isolated and purified using the phenol-chloroform method. The mitochondrial genome of *T. drosophilae* was sequenced using Illumina HiSeq X Ten system with the strategy of 150 paired-ends reading. It was further annotated using the Geneious 11.0.4 version and MITOS Web Server (Bernt et al. 2013).

The length of the complete mitochondrial genome of *T. drosophilae* is 16,375 bp, and it contains 37 genes which includes 13 protein-coding genes (PCGs), 22 transfer RNA genes (tRNAs), 2 ribosomal RNA genes (rRNAs), and a putative control region (CR). Further analysis revealed that 24 genes were encoded on the majority stand, and the remaining 13 genes were encoded on the minority strand. Three main rearrangement events of tRNA clusters were found in the sequenced region compared with the putative ancestral arrangement of insects, corresponding to the Q-M-I, W-C, and D-K patterns. Besides, the *trnV* gene was transposed to the upstream of the *rrnS* gene from the original region of rrnS-trnV-A + T-rich region and *trnL2* was transposed to the upstream of *nad1* gene from the original region of cox1-trnL2-cox2. Moreover, all of PCGs and tRNAs showed a same order and direction as the putative ancestral arrangement of insects except for *nad4L*, which has an inversion in its original position (Wei et al. 2014). The overall base composition is 44.9% for A, 6.4% for G, 5.6% for C, and 43.1% for T, with an A + T content of 88.0%. Three start codons for PCGs were used: ATT (*nad1*, *nad3*, *nad4*, *nad4L*, *nad6*, *cox2* and *atp8*); ATG (*atp6*, *cox3* and *cob*); ATA (*cox1*, *nad2*, and *nad5*), and all 13 PCGs used a TAA stop codon. The 22 tRNAs genes varied from 51 to 69 bp in length, and the secondary structure of tRNAs was a typical clover-leaf structure as with other insects. The *rrnL* was located between *trnL1* and *trnY*, whereas *rrnS* was between *trnY* and *trnV*. The length of *rrnL* and *rrnS* was 1271 and 757 bp, respectively.

Approximately 50 hymenopterous parasitoid species have been reported attacking various *drosophilid* species worldwide, the majority of which are larval parasitoids in the families Braconidae and Figitidae and pupal parasitoids in the families Diapriidae and Pteromalidae (Fleury et al. 2009; Cancino et al. 2015; Miller et al. 2015). We perfomed a phylogenetic analysis of *T. drosophilae* with some other parasitoids from the above four families (Oliveira et al. 2008, 2016; Li et al. 2016; Tang et al. 2019; Zhang et al. 2020). The sequences were aligned by using MAFFT v7.271, and the phylogenetic tree was constructed by CIPRES (https://www.phylo.org/) using RAxML-HPC2 on XSEDE with bootstrap 1000 and MrBayes on XSEDE (Figure 1). Phylogenetic analysis showed that *T. drosophilae* is closely related to *Ismarus sp.*, another species of Diapriidae. It is obvious that the four families are grouped in different clusters. This study would further clarify our understanding of the phylogenetic relationship of the Diapriidae family.

## Data Availability

The data that support the findings of this study are openly available in GenBank of NCBI at https://www.ncbi.nlm.nih.gov, reference number MN966974. Phylogenetic relationships among four parasitoid families that all parasitize *drosophilid* species inferred from nucleotides of 13 PCGs and two rRNAs using Bayesian and Maximum-likelihood (ML) methods (GenBank accession numbers provided). The Bayesian posterior probabilities (PP) and bootstrap support (BS) are marked besides the nodes. *Drosophila incompta* were set as outgroup.
